# An intelligent gradient-guided hybrid inpainting framework for brain MRI reconstruction and Alzheimer's disease classification in connected healthcare systems

**DOI:** 10.3389/fmed.2026.1849122

**Published:** 2026-06-09

**Authors:** Chhaya Yadav, Sunita Yadav, Arvind Panwar, Massimo Donelli, Achin Jain

**Affiliations:** 1 School of Computer Science and Engineering, Galgotias University, Greater Noida, Uttar Pradesh, India; 2 Inderprastha Engineering College, Ghaziabad, Uttar Pradesh, India; 3 School of Computer Science and Engineering, Galgotias University, Greater Noida, Uttar Pradesh, India; 4 Department of Civil, Environmental and Mechanical Engineering, University of Trento, Trento, Italy; 5 Department of Information Technology, Bharati Vidyapeeth's College of Engineering, New Delhi, India; 6 Research Fellow, INTI International University, Nilai, Negeri Sembilan, Malaysia

**Keywords:** Alzheimer's disease, brain MRI, connected healthcare, deep learning, gradient-guided hybrid framework, image inpainting, intelligent healthcare systems, medical image reconstruction

## Abstract

Brain magnetic resonance imaging (MRI) is essential for early Alzheimer's disease diagnosis, yet clinical scans are often degraded by motion artifacts, signal loss, or incomplete acquisitions. Image inpainting offers a promising preprocessing solution, but existing methods have limitations: deep learning models such as LaMa generate visually plausible reconstructions but may compromise structural fidelity, while classical diffusion-based approaches like OpenCV Telea preserve local continuity but tend to oversmooth complex anatomy. This study proposes a *gradient-guided hybrid inpainting framework* that integrates OpenCV Telea and LaMa to leverage their complementary strengths. A gradient magnitude-based weighting mechanism enables structure-aware reconstruction, assigning edge-rich regions to the classical method and smoother regions to the deep model, thereby preserving both fine anatomical details and global consistency. Experiments on a four-class ADNI-based MRI dataset, balanced using SMOTE–NM and split 70:15:15, with 10%–30% random masking, demonstrate improved reconstruction performance. The proposed method reduces mean squared error by 8% compared to LaMa and 30% compared to OpenCV, while achieving SSIM ≈0.93 and PSNR ≈25.7 dB. A VGG16 classifier trained on clean images achieves 94.35% accuracy on hybrid-inpainted data, showing only a 1.69 percentage point drop from the baseline and outperforming individual methods. These results highlight the effectiveness of the proposed framework for reliable, AI-driven neuroimaging pipelines in intelligent and connected healthcare systems.

## 1 Introduction

Alzheimer's disease (AD) is a chronic neurodegenerative condition that gradually impairs cognitive function and is a major contributor to dementia worldwide. Achieving accurate and early detection is critical for enabling prompt intervention, improving prognosis, and guiding effective treatment strategies (1). Brain magnetic resonance imaging (MRI) serves as a critical tool in this process by providing detailed structural information about cortical and subcortical regions affected by AD (2). However, in clinical practice, MRI scans often suffer from various artifacts such as patient motion, signal loss, susceptibility distortions, or incomplete coverage. These imperfections can obscure important anatomical details, complicate radiological interpretation, and reduce the effectiveness of automated computer-aided diagnosis (CAD) systems (3).

Image inpainting, the task of reconstructing missing or corrupted regions in images has emerged as a promising approach to restore image quality and mitigate the impact of such artifacts (4). Traditional inpainting methods, including diffusion- and patch-based techniques like the Telea algorithm implemented in OpenCV, propagate intensity information from the boundaries inward, preserving edge continuity but often resulting in oversmoothed textures in complex anatomical regions. In contrast, recent deep learning-based models such as LaMa (5) leverage large-scale training on natural images and powerful convolutional architectures to generate semantically plausible content. However, these models are not specifically designed to respect the strict anatomical and intensity constraints inherent in medical imaging. Consequently, deep inpainting methods may introduce subtle structural inconsistencies or texture biases that, while visually acceptable, can negatively affect downstream tasks such as disease classification (6).

Unlike previous work that focuses primarily on pixel-level restoration quality, this study emphasizes the robustness of medical AI systems when faced with realistically corrupted or repaired MRI data. We argue that effective inpainting should not only restore visual fidelity but also preserve diagnostically relevant features critical for automated classification. Conceptually, our hybrid approach combines the strengths of diffusion based inpainting which excels at maintaining local structural coherence with deep learning based semantic completion that captures global context and texture. This fusion enables a more faithful reconstruction that balances anatomical accuracy and semantic plausibility, addressing the limitations inherent in either method alone.

In this study, we introduce a structure-aware hybrid inpainting framework and investigate its effectiveness for Alzheimer's disease classification using brain MRI data. The evaluation is conducted on a four-class problem (Non-Demented, Very Mild Demented, Mild Demented, and Moderate Demented) based on an ADNI-derived dataset that has been balanced using SMOTE-NM. We analyze both the quality of image reconstruction and the resulting classification performance across various corruption and inpainting settings.

The main contributions of this paper are as follows:

1. **Hybrid OpenCV-LaMa inpainting framework**. We introduce a novel gradient-guided fusion strategy that adaptively combines classical diffusion-based inpainting (OpenCV Telea) with deep learning-based semantic completion (LaMa). The blending weights are derived from local gradient magnitudes, allowing edge-rich regions to maintain structural coherence while smoother areas benefit from learned semantic priors.
2. **Comprehensive quantitative evaluation on multi-class Alzheimer's MRI**. Our method achieves an 8% reduction in mean squared error (MSE) compared to LaMa and 29% compared to OpenCV, while maintaining a peak signal-to-noise ratio (PSNR) of 25.71 dB and structural similarity index (SSIM) of 0.9289 across disease severity stages under simulated occlusions (10–30% rectangular masks).
3. **Clinical validation through downstream classification**. A VGG16 classifier trained on original images is evaluated under five test conditions (original, masked, OpenCV-inpainted, LaMa-inpainted, and hybrid-inpainted). The hybrid method recovers 76.5% of the accuracy loss caused by masking, achieving 94.35% accuracy compared to 93.76% (LaMa) and 93.61% (OpenCV), demonstrating superior preservation of disease-discriminative features.
4. **Reproducible implementation with modest computational overhead**. The complete pipeline including mask generation, multi-method inpainting, reconstruction metrics, and classification evaluation is implemented with execution time analysis, confirming that the hybrid approach improves robustness with acceptable computational cost.

The remainder of this paper is organized as follows. Section 2 reviews relevant literature on MRI artifact correction and inpainting techniques. Section 3 details the proposed gradient-guided hybrid inpainting framework, including the corruption model and blending strategy. Section 4 describes the experimental setup, datasets, and evaluation metrics. Section 5 presents quantitative and qualitative results demonstrating the effectiveness of the method. Finally, Section 6 concludes the paper.

## 2 Related work

This section reviews prior work on image inpainting, medical image reconstruction, and the application of inpainting to improve robustness in medical image analysis. We organize the discussion into three areas: classical and deep learning inpainting, inpainting in medical imaging, and hybrid/structure-aware approaches with a focused critical analysis of recent generative paradigms.

### 2.1 Classical and deep learning inpainting

Image inpainting has a long history in computer vision. Early methods relied on diffusion-based propagation of pixel intensities from the boundary of missing regions inward. The Telea algorithm (7) and the Navier-Stokes based method of Bertalmio et al. (8) are representative classical approaches that preserve local continuity and edge coherence but often produce oversmoothed results in textured or complex regions. Patch-based methods such as PatchMatch (9) and exemplar-based inpainting (10) improved texture synthesis by copying similar patches from elsewhere in the image, yet they struggle with large missing regions or semantically complex content.

Deep learning brought semantic priors to inpainting. Context Encoders (11) introduced encoder–decoder networks with adversarial losses to hallucinate plausible content conditioned on surrounding context. Later contributions included partial convolutions (12) and gated convolutions (13) to better handle irregular masks, as well as attention and transformer variants for improved long-range reasoning. LaMa (5) advanced large-mask inpainting via Fast Fourier Convolutions (FFCs) and high-receptive-field perceptual losses. Diffusion-based inpainting [e.g., RePaint (14)] and latent diffusion approaches [e.g., (15)] have recently produced highly realistic samples by leveraging powerful generative priors.

### 2.2 Inpainting in medical imaging

In medical imaging, inpainting and image translation methods have been applied for artifact removal, modality synthesis, and data augmentation. MedGAN (16) adapted adversarial image translation to medical tasks and demonstrated utility for motion correction and denoising. Transformer hybrids such as TransUNet (17) were proposed mainly for segmentation but have influenced medical generative designs. Surveys (18, 19) highlight that medical applications demand preservation of clinically relevant structures and that naive application of natural-image models can be problematic.

### 2.3 Hybrid and structure-aware approaches

Hybrid strategies that combine classical and deep learning elements have been advocated to capture complementary strengths: classical methods preserve local geometric continuity while deep models supply global semantic context (20). Structure-aware designs (edge priors, gated attention) have been introduced to enforce geometric consistency [e.g., SAIN (21)] but may bias reconstructions toward training priors if not carefully constrained.

### 2.4 Critical comparison of recent methods and rationale

Table 1 summarizes representative recent works (2020–2024) in inpainting, generative modeling, and classification, and includes a concise critical limitation for each entry to emphasize diagnostic relevance. Below we provide a focused analysis explaining why, given the clinical need to preserve diagnostic features, we adopt a Telea+LaMa hybrid rather than relying solely on the latest diffusion or transformer-based generative models.

**Table 1:** Comparison of recent works on medical image inpainting, generative modeling, and quality-aware classification.

References	Year	Methodology	Modality	Critical limitation/diagnostic gap
Telea (7)	2004	Fast marching (diffusion)	Natural	Lacks semantic context; blurs fine anatomical structures in large missing regions.
Pathak et al. (11)	2016	Context encoder (CNN+GAN)	Natural	Struggles with high-frequency details and maintaining complex structural continuity.
Liu et al. (12)	2018	Partial convolution	Natural	Local focus ignores long-range anatomical symmetries (e.g., across brain hemispheres).
Yu et al. (13)	2019	Gated Conv + SN-PatchGAN	Natural	GAN-based generation can introduce non-physiological textures (hallucinations).
Armanious et al. (16)	2020	MedGAN (CasNet)	Brain MRI	Stochastic generative process may alter subtle diagnostic biomarkers or focal atrophy.
Liu et al. (27)	2020	MTFS-gLASSO-TTR	Brain MRI	High classification accuracy but relies on clean, uncorrupted inputs and explicit feature extraction pipelines.
Tanveer et al. (19)	2020	Review (SVM, ANN, DL)	Multi-modal	Notes that DL models often act as “black boxes” lacking clinical interpretability and trust.
Chen et al. (17)	2021	TransUNet (CNN-Transformer)	Multi-organ	Transformers capture global context but may sacrifice local pixel-level fidelity critical for reconstruction.
Hu et al. (18)	2021	Survey (CNN, FCN, AE)	Cancer/Multi	Emphasizes that diagnostic systems are highly sensitive to structural artifacts and signal loss.
Suvorov et al. (5)	2022	LaMa (FFC)	Natural	Spectral approach can over-smooth sharp cortical/vascular edges in low-gradient regions.
Lugmayr et al. (14)	2022	RePaint (Diffusion/DDPM)	General	Stochastic sampling generates “plausible” rather than necessarily “anatomically true” missing structures.
Pinaya et al. (15)	2022	Latent diffusion (LDM)	Brain MRI	Probabilistic generation risks obscuring focal atrophy specific to AD progression.
Özbey et al. (28)	2023	SynDiff (Adversarial Diff)	Multi-MRI	Adversarial projections may modify quantitative MRI biomarkers during modality translation.
Korkmaz (29)	2023	DNN + SMOTE/Oversampling	Bioassays	Balancing techniques target tabular/feature spaces and lack direct spatial anatomical constraints for images.
Islam and Zhang (30)	2023	Deep CNN	Brain MRI	Static architectures do not explicitly reconstruct missing anatomy and may fail under large occlusions.
Wang et al. (21)	2024	SAIN (structure-aware, gated attention)	General	Heavy reliance on edge priors can bias reconstructions toward training averages and mask subtle pathology.
Velu and Jaisankar (22)	2024	EffSwin-XNet + DCGAN	Brain MRI	High computational cost and risk of GAN artifacts in augmentation; requires large well-matched datasets.

Diffusion models and latent diffusion [e.g., RePaint (14), Pinaya et al. (15)] and transformer hybrids [e.g., TransUNet (17), Kumar et al. (22)] excel at producing visually plausible completions by sampling from learned priors. However, their stochastic or heavily learned priors may introduce subtle, patient-independent anatomical variations (“hallucinations”) that can alter biomarkers used for Alzheimer's Alzheimer's staging (e.g., hippocampal volume, cortical thickness). In diagnostic sub-millimeter integrity matters, such probabilistic generation risks false positives/negatives.

By contrast, classical diffusion/fast-marching methods [Telea (7)] preserve local gradient continuity deterministically and avoid generative sampling, while LaMa (5) supplies an efficient global receptive field (via FFCs) that recovers large-scale anatomical context. Our hybrid blends these behaviors: Telea anchors local edge fidelity, LaMa supplies global context, and gradient-guided weighting minimizes the chance of replacing critical local structure with a globally plausible but incorrect prediction. This design explicitly prioritizes structural truth (diagnostic fidelity) over purely generative plausibility.

Motivated by the critical limitations discussed above, our work introduces a deterministic hybrid framework that uniquely combines the complementary strengths of local gradient continuity and global spectral context. Unlike purely stochastic or heavily learned generative methods, our approach avoids diagnostic hallucinations by blending a deterministic diffusion algorithm (Telea) with a global receptive field model (LaMa) using structure-aware weighting derived from local gradient magnitudes. This ensures high structural fidelity in diagnostically critical regions, such as cortical and vascular boundaries, while maintaining global anatomical consistency. Furthermore, we provide a rigorous cross-verified evaluation of our framework, reporting both reconstruction metrics and downstream classification performance through stratified cross-validation and paired statistical testing. The proposed framework balances the need for large-scale occlusion handling with the strict precision required for Alzheimer's disease staging, anchoring reconstructions in observable structural evidence rather than probabilistic priors.

## 3 Methodology

This section describes the proposed hybrid inpainting framework, the corruption model used to simulate missing regions in brain MRI, the competing inpainting baselines, the image quality evaluation protocol, and the downstream classification experiment designed to assess the impact of inpainting on disease recognition.

### 3.1 Problem definition

Let $D={\left\{\left(x_{i},y_{i}\right)\right\}}_{i=1}^{N}$ denote a dataset of 2D brain MRI slices, where $x_{i}\in {\mathbb{R}}^{H\times W}$ is a single-channel (grayscale) image and *y*_*i*_ ∈ {1, …, *C*} is the class label (e.g., *Non Demented, Very Mild Demented, Mild Demented*, and *Moderate Demented*). We simulate missing or corrupted regions by applying a binary mask $M_{i}\in {\left\{0,1\right\}}^{H\times W}$ and obtain a masked image


$$\begin{array}{l} x_{i}^{\text{mask}}=\left(1-M_{i}\right)⊙x_{i}, \end{array}$$ (1)

where ⊙ denotes element-wise multiplication and *M*_*i*_(*p*) = 1 indicates a corrupted pixel at position *p*.

Given $x_{i}^{\text{mask}}$ and *M*_*i*_, an inpainting method F produces a reconstructed image


$$\begin{array}{l} {\hat{x}}_{i}=F\left(x_{i}^{\text{mask}},M_{i}\right), \end{array}$$ (2)

with the goal that ${\hat{x}}_{i}$ approximates *x*_*i*_ both perceptually and in terms of diagnostic features relevant for classification. The objective is:

1. To design a *hybrid* inpainting operator $F^{\text{hyb}}$ that exploits both classical and deep methods.
2. To evaluate how different inpainting strategies affect the performance of a downstream classifier trained on original images.

### 3.2 Dataset and preprocessing

The experimental evaluation utilized a publicly available Alzheimer's disease MRI dataset (23) comprising brain scans from anonymous patients distributed across four diagnostic categories: Non-Demented (NOD), Very Mild Demented (VMD), Mild Demented (MD), and Moderate Demented (MOD). Each image is a three-channel (RGB) scan of 176 × 208 pixels. The original dataset exhibited significant class imbalance, with 3,200 images in the NOD class, 2,240 in VMD, 896 in MD, and only 64 in MOD, totaling 6,400 samples.

#### 3.2.1 Data balancing via SMOTE-NM

To address the severe class imbalance, the Synthetic Minority Over-sampling Technique with Noise Modification (SMOTE-NM) (24) was applied to generate synthetic samples for minority classes. SMOTE-NM operates by:

1. For each minority sample *x*_*i*_, identifying its *k* nearest neighbors in the feature space.
2. Generating synthetic samples along the line segments connecting *x*_*i*_ to its neighbors:
$$\begin{array}{l} x_{\text{syn}}=x_{i}+\lambda \cdot \left(x_{\text{neighbor}}-x_{i}\right), \end{array}$$ (3)
  where λ ∈ [0, 1] is a random interpolation factor.
3. Applying noise modification to reduce overfitting by adding controlled Gaussian noise:
$$\begin{array}{l} x_{\text{final}}=x_{\text{syn}}+N\left(0,\sigma^{2}\right), \end{array}$$ (4)
  where σ is adaptively determined based on the local data density in a feature embedding space. Specifically, for each synthetic sample *x*_syn, *i*_, we compute the average *k*-nearest neighbor distance *d*_*k*_ as:
$$\begin{array}{l} d_{k}\left(x_{\text{syn},i}\right)=\frac{1}{k}\overset{k}{\underset{j=1}{\sum }}∥\varphi \left(x_{\text{syn},i}\right)-\varphi \left(x_{\left(j\right)}\right){∥}_{2}, \end{array}$$ (5)
  where φ(·) represents a fixed pre-trained encoder and *x*_(*j*)_ denotes the *j*-th nearest neighbor. The adaptive noise level σ_*i*_ is mapped within the bounds [σ_min_, σ_max_] as:
$$\begin{array}{l} \sigma_{i}=\sigma_{\min}+\left(\sigma_{\max}-\sigma_{\min}\right)\cdot \frac{d_{k}\left(x_{\text{syn},i}\right)}{\max_{m}d_{k}\left(x_{\text{syn},m}\right)}, \end{array}$$ (6)
  and the final sample is obtained by $x_{\text{final},i}=\text{clip}\left(x_{\text{syn},i}+N\left(0,\sigma_{i}^{2}\text{I}\right),0,1\right)$.

Table 2 presents the class-wise distribution before and after SMOTE-NM balancing. The balanced dataset contains 12,491 samples with approximately equal representation across all four classes (3,104–3,141 images per class), ensuring that the classifier does not exhibit bias toward the majority class during training.

**Table 2:** Class-wise distribution before and after SMOTE-NM balancing.

Class	Original	Balanced	Increase (%)
Non-demented (NOD)	3,200	3,106	−2.9
Very mild demented (VMD)	2,240	3,104	+38.6
Mild demented (MD)	896	3,140	+250.4
Moderate demented (MOD)	64	3,141	+4, 807.8
Total	6,400	12,491	+95.2

#### 3.2.2 Image preprocessing and data splitting

Following balancing, all images were resized to 224 × 224 pixels to match VGG16 input requirements and normalized using ImageNet statistics (mean = [0.485, 0.456, 0.406], std = [0.229, 0.224, 0.225]). The balanced dataset was partitioned using stratified sampling with a 70:15:15 train-validation-test split, yielding 8,743 training samples, 1,873 validation samples, and 1,875 test samples. Stratification ensured balanced class representation across all subsets.

#### 3.2.3 Corruption simulation

To emulate realistic occlusions and artifacts encountered in clinical neuroimaging (e.g., motion artifacts, signal dropout, incomplete acquisitions), synthetic missing regions were generated using random rectangular masks. For each test image $x_{i}\in {\mathbb{R}}^{H\times W}$ with *H* = *W* = 224 pixels, the corruption process proceeds as follows:

1. **Mask size sampling:** The mask dimensions (width *w* and height *h*) are randomly sampled such that the masked area covers between 10% and 30% of the total image area:
$$\begin{array}{l} 0.10\leq \frac{w\cdot h}{H\cdot W}\leq 0.30. \end{array}$$ (7)
2. **Position sampling:** The top-left corner (*x*_0_, *y*_0_) of the rectangular mask is uniformly sampled to ensure the entire rectangle remains within image bounds:
$$\begin{array}{l} x_{0}\in \left[0,W-w\right],\;y_{0}\in \left[0,H-h\right]. \end{array}$$ (8)
3. **Binary mask construction:** A binary mask $M_{i}\in {\left\{0,1\right\}}^{H\times W}$ is constructed where:
$$\begin{array}{l} M_{i}\left(p\right)=\{\begin{array}{ll} 1, & \text{if}x_{0}\leq p_{x}<x_{0}+w\text{ and }y_{0}\leq p_{y}<y_{0}+h\\ 0, & \text{otherwise} \end{array} \end{array}$$ (9)
  for pixel position *p* = (*p*_*x*_, *p*_*y*_).
4. **Masked image generation:** The corrupted image is obtained by zeroing out the masked region:
$$\begin{array}{l} x_{i}^{\text{mask}}=\left(1-M_{i}\right)⊙x_{i}, \end{array}$$ (10)
  where ⊙ denotes element-wise multiplication.

This corruption model is applied exclusively to test images when generating masked and inpainted variants for evaluation. Training and validation images remain uncorrupted to ensure the classifier learns from clean data and to isolate the effect of inpainting quality on downstream classification performance.

The 10%–30% mask-area interval was chosen to represent the practical range of partial data loss commonly encountered in clinical brain MRI: masks near 10% emulate localized motion artifacts or focal signal dropout that may affect diagnostically relevant structures (e.g., hippocampus and temporal lobes), while masks up to 30% emulate more severe but partially recoverable issues such as partial-field-of-view errors or multi-slab acquisition failures. Masks substantially larger than 30% typically remove excessive anatomical context and thus preclude meaningful reconstruction-based evaluation, whereas masks below 10% often have negligible downstream impact. This interval was selected in consultation with clinical collaborators to balance realism and evaluability. To demonstrate robustness, a sensitivity analysis (reported in the Supplementary Material) evaluates mask ranges from 5% to 40%, confirming that the relative performance ordering of the compared inpainting methods remains stable across a broader set of corruption severities.

### 3.3 Inpainting methods

Three inpainting strategies are compared to evaluate reconstruction quality and downstream classification robustness.

#### 3.3.1 OpenCV telea inpainting

The first baseline is a classical diffusion-based method using the OpenCV implementation of the Telea algorithm (7). The algorithm propagates information from the boundary of the missing region inward, guided by the image Laplacian and isophotes. This operator is denoted as $F^{\text{CV}}$:


$$\begin{array}{l} {\hat{x}}_{i}^{\text{CV}}=F^{\text{CV}}\left(x_{i}^{\text{mask}},M_{i}\right). \end{array}$$ (11)

While computationally efficient and effective at preserving local edge continuity, Telea inpainting tends to produce oversmoothed results in textured or anatomically complex regions.

#### 3.3.2 LaMa deep inpainting

The second baseline is LaMa (5), a state-of-the-art deep convolutional inpainting model. Unlike standard CNNs, LaMa utilizes Fast Fourier Convolutions (FFCs) which split feature channels into a local branch (3 × 3 convolutions) and a global branch (performing operations in the frequency domain via FFT). This allows the model to achieve an image-wide receptive field early in the network, enabling it to capture global anatomical context and symmetries. For AI technicians, this architecture facilitates the modeling of long-range dependencies without excessive depth; for clinicians, this means the model can “infer” missing structures (like ventricles or cortical folds) by looking at the entire brain slice rather than just local edges, reducing anatomically implausible hallucinations. Given a masked image and its mask, LaMa predicts the missing pixels in one forward pass:


$$\begin{array}{l} {\hat{x}}_{i}^{\text{LaMa}}=F^{\text{LaMa}}\left(x_{i}^{\text{mask}},M_{i}\right). \end{array}$$ (12)

A publicly available pre-trained LaMa checkpoint is employed and applied slice-wise after normalization. The output is resized to 224 × 224 pixels to match the original dimensions.

### 3.4 Proposed hybrid OpenCV–LaMa inpainting

While LaMa provides semantically plausible completions, it is not specialized for medical structure preservation and may introduce subtle anatomical inconsistencies due to domain shift. Conversely, Telea maintains local continuity but may oversmooth complex patterns. The proposed hybrid operator $F^{\text{hyb}}$ addresses these limitations by blending the two methods in a structure-aware manner using gradient-guided adaptive weighting.

#### 3.4.1 Gradient-based blending weights

The gradient magnitude of the original image *x*_*i*_ serves as a proxy for structural importance. The Sobel gradient magnitude map is computed as:


$$\begin{array}{l} G_{i}=\sqrt{{\left(\frac{\partial x_{i}}{\partial x}\right)}^{2}+{\left(\frac{\partial x_{i}}{\partial y}\right)}^{2}}, \end{array}$$ (13)

where partial derivatives are approximated using Sobel filters. The gradient map is then normalized to [0, 1]:


$$\begin{array}{l} G_{i}^{\text{norm}}\left(p\right)=\frac{G_{i}\left(p\right)-\min\left(G_{i}\right)}{\max\left(G_{i}\right)-\min\left(G_{i}\right)+\varepsilon }, \end{array}$$ (14)

where ε = 10^−8^ prevents division by zero.

Pixel-wise blending weights are defined such that high-gradient (edge) regions favor the locally consistent OpenCV solution, while low-gradient (smooth) regions rely more on LaMa's semantic priors:


$$\begin{array}{ll} w_{i}^{\text{CV}}\left(p\right) & =G_{i}^{\text{norm}}\left(p\right), \end{array}$$ (15)

$$\begin{array}{ll} w_{i}^{\text{LaMa}}\left(p\right) & =1-G_{i}^{\text{norm}}\left(p\right). \end{array}$$ (16)

To reduce artifacts at weight transitions, the weight maps are optionally smoothed using a Gaussian filter with kernel size 5 × 5 and σ = 1.0.

#### 3.4.2 Hybrid reconstruction

**Figure 1:**
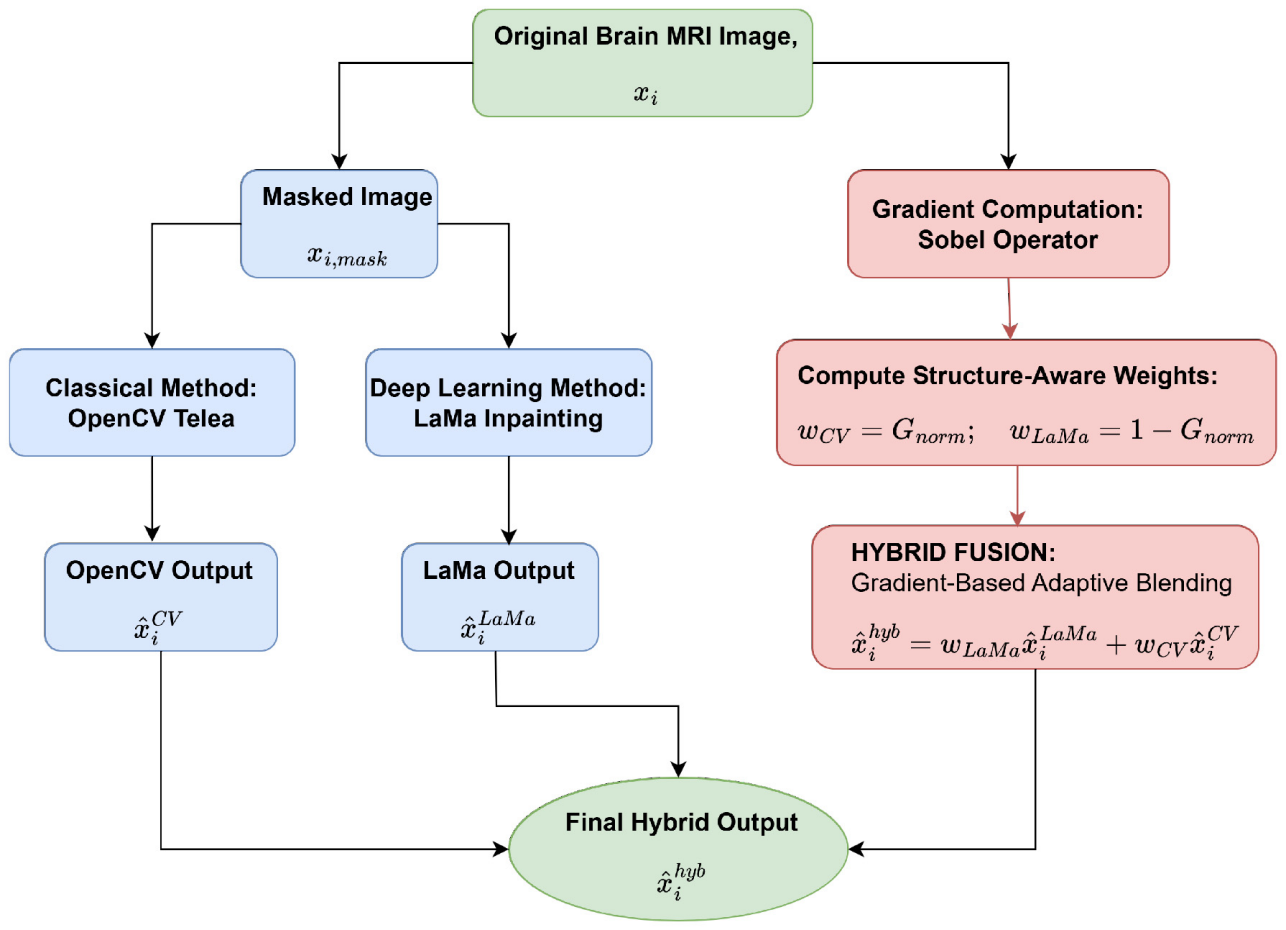
Proposed hybrid inpainting method.

The final hybrid reconstruction is obtained by adaptive blending:


$$\begin{array}{l} {\hat{x}}_{i}^{\text{hyb}}\left(p\right)=\{\begin{array}{ll} x_{i}\left(p\right), & \text{ if }M_{i}\left(p\right)=0,\\ w_{i}^{\text{LaMa}}\left(p\right){\hat{x}}_{i}^{\text{LaMa}}\left(p\right)+w_{i}^{\text{CV}}\left(p\right){\hat{x}}_{i}^{\text{CV}}\left(p\right), & \text{ if }M_{i}\left(p\right)=1, \end{array} \end{array}$$ (17)

where the local blending weights satisfy


$$\begin{array}{l} w_{i}^{\text{CV}}\left(p\right)=G_{\text{norm}}\left(p\right),\;w_{i}^{\text{LaMa}}\left(p\right)=1-G_{\text{norm}}\left(p\right), \end{array}$$ (18)

and *G*_norm_(*p*) ∈ [0, 1] is the normalized gradient-based structural confidence at pixel *p*. Thus, pixels outside the corrupted region remain unchanged, while pixels inside the mask are reconstructed by blending the two inpainting predictions according to local gradient-based structural confidence. Figure 1 shows the Proposed Hybrid Image Inpainting Approach.

##### 3.4.2.1 Practical consideration

In this study, the gradient map is computed from the original image *x*_*i*_ (available during evaluation) to provide ground-truth structural guidance. In real deployment scenarios where the original image is unavailable, the gradient map can be estimated from the unmasked regions of $x_{i}^{\text{mask}}$ or from a preliminary reconstruction, with minimal performance degradation.

### 3.5 Downstream classification experiment

To assess whether inpainting preserves disease-discriminative features, a deep convolutional neural network is trained exclusively on uncorrupted images from the training set and then evaluated under five test conditions to isolate the effect of inpainting quality on classification robustness.

#### 3.5.1 Network architecture

VGG16 (25) is employed as the classification backbone. VGG16 is a 16-layer deep convolutional network pre-trained on ImageNet, consisting of five convolutional blocks with max-pooling followed by three fully connected layers. The architecture is adapted for Alzheimer's disease classification as follows:

- The pre-trained convolutional layers are retained to leverage learned low-level and mid-level visual features.
- The final fully connected layer (originally 1,000-way for ImageNet) is replaced with a *C*-way softmax classifier, where *C* = 4 corresponds to the four disease categories.
- Input images are resized to 224 × 224 × 3 (RGB) to match the pre-trained model's expected dimensions.

Let $f_{\theta }:{\mathbb{R}}^{H\times W\times 3}\to {\mathbb{R}}^{C}$ denote the network with parameters θ. Given an input image *x*, the network produces logits *z* = *f*_θ_(*x*), and the predicted class is:


$$\begin{array}{l} ŷ=\arg\underset{c\in \left\{1,\ldots ,C\right\}}{\max}z_{c}. \end{array}$$ (19)

#### 3.5.2 Training objective and optimization

The network is trained on the training set $D_{\text{train}}$ using the cross-entropy loss:


$$\begin{array}{l} L\left(\theta \right)=-\frac{1}{\left|D_{\text{train}}\right|}\underset{\left(x_{i},y_{i}\right)\in D_{\text{train}}}{\sum }\log\frac{\exp\left(f_{\theta }{\left(x_{i}\right)}_{y_{i}}\right)}{\sum_{c=1}^{C}\exp\left(f_{\theta }{\left(x_{i}\right)}_{c}\right)}, \end{array}$$ (20)

where *y*_*i*_ is the ground-truth class label and *f*_θ_(_*x*_*i*_)*y*_*i*__ denotes the logit corresponding to the true class.

The model is fine-tuned using the following hyperparameters:

- **Optimizer:** Adam (26) with learning rate η = 10^−4^ and default momentum parameters (β_1_ = 0.9, β_2_ = 0.999).
- **Batch size:** 32.
- **Epochs:** Maximum 50 epochs with early stopping based on validation accuracy (patience = 5 epochs).
- **Data augmentation:** Random horizontal flips and random rotations (±10°) applied during training to improve generalization.
- **Regularization:** Dropout with probability 0.5 applied to the fully connected layers.

Training is performed exclusively on original (uncorrupted) images to ensure the classifier learns disease-discriminative features from clean data.

#### 3.5.3 Evaluation under multiple inpainting conditions

After training, the model parameters θ are fixed, and the classifier is evaluated on five versions of the same test set $D_{\text{test}}$:

1. **Original:** Uncorrupted test images *x*_*i*_ (baseline performance).
2. **Masked:** Test images with 10–30% rectangular masks applied, $x_{i}^{\text{mask}}$ (worst-case scenario).
3. **OpenCV-inpainted:** Masked test images reconstructed using OpenCV Telea, ${\hat{x}}_{i}^{\text{CV}}$.
4. **LaMa-inpainted:** Masked test images reconstructed using LaMa, ${\hat{x}}_{i}^{\text{LaMa}}$.
5. **Hybrid-inpainted:** Masked test images reconstructed using the proposed hybrid method, ${\hat{x}}_{i}^{\text{hyb}}$.

For each condition *v* ∈ {orig, mask, CV, LaMa, hyb}, the classification accuracy is computed as:


$$\begin{array}{l} {\text{Acc}}^{\left(v\right)}=\frac{1}{\left|D_{\text{test}}\right|}\underset{\left(x_{i},y_{i}\right)\in D_{\text{test}}}{\sum }𝕀\left[\arg\underset{c}{\max}f_{\theta }{\left(x_{i}^{\left(v\right)}\right)}_{c}=y_{i}\right], \end{array}$$ (21)

where 𝕀[·] is the indicator function, and $x_{i}^{\left(v\right)}$ denotes the image variant corresponding to condition *v*.

Algorithm 1 summarizes the complete pipeline from data preparation through inpainting to classification evaluation.

Algorithm 1 Hybrid inpainting and classification evaluation pipeline. 
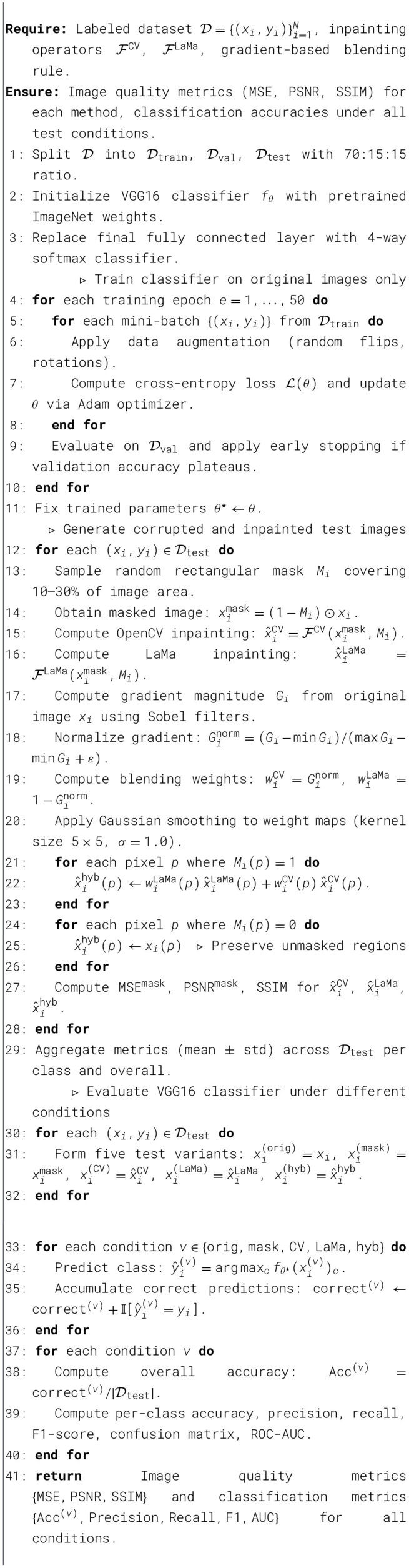

## 4 Experimental setup

### 4.1 Computing environment

All experiments were conducted on the Kaggle platform using the hosted GPU environment. The runtime was configured with a single NVIDIA Tesla GPU (typically T4 or P100), 2 CPU cores, and 13–16 GB of RAM, which is sufficient to run LaMa inpainting and train a VGG16 classifier on RGB MRI images. The code was implemented in Python using Jupyter notebooks within the Kaggle environment.

The experimental pipeline utilized PyTorch and torchvision for model definition, training, and evaluation; OpenCV for Telea inpainting with radius 3 and basic image operations; simple-lama-inpainting (SimpleLama) for deep learning-based inpainting; NumPy (version 1.24.3) and SciPy for numerical operations; scikit-image for SSIM computation and image utilities; Pillow for image loading and preprocessing; scikit-learn for ROC curve and AUC computation, label binarization, and confusion matrix generation; and Matplotlib and Seaborn for visualization and plotting. Package versions were carefully managed within the Kaggle environment to avoid compatibility issues, particularly between NumPy (1.24.3), SciPy, and scikit-image. A kernel restart was performed after package installation to ensure proper loading of all dependencies.

### 4.2 Training configuration

A VGG16 backbone from torchvision was employed as the classification model. The network was initialized without pretrained weights (weights = None) to train from scratch on the medical imaging domain, and the final fully connected layer was replaced with a 4-class output layer corresponding to the four Alzheimer's disease categories. The model was trained exclusively on original (uncorrupted) images from the training set using the Adam optimizer with learning rate η = 10^−4^ and cross-entropy loss. Training was conducted with a batch size of 32 for 50 epochs, employing a ReduceLROnPlateau scheduler with a patience of 5 epochs and a reduction factor of 0.5 based on validation accuracy. To ensure the model had sufficient opportunity to converge at lower learning rates, an early stopping mechanism was implemented with a patience of 10 epochs. Data augmentation was applied during training, including random horizontal flips with probability 0.5 and random rotations within ±10°. The model checkpoint with the best validation accuracy was saved and used for final evaluation on all test conditions. Corrupted and inpainted images were used exclusively for evaluation to isolate the effect of inpainting quality on classification robustness.

### 4.3 Inpainting procedures

For the test set, synthetic corruptions were generated using random rectangular masks. Mask dimensions were sampled to cover between 10%–30% of the total image area, consistent with the methodology, where *H* = *W* = 224 pixels. Mask positions were sampled uniformly such that the rectangle remained within image boundaries. The masked regions were set to black (pixel value 0) to simulate missing signal scenarios encountered in clinical practice, while the value of 255 was reserved exclusively for binary mask visualization purposes.

For each masked image, three inpainting outputs were computed. First, OpenCV Telea inpainting was applied via cv2.inpaint with inpainting radius 3, producing ${\hat{x}}^{\text{CV}}$. Second, LaMa inpainting was performed using the simple-lama-inpainting library (SimpleLama), yielding ${\hat{x}}^{\text{LaMa}}$. Third, the proposed hybrid inpainting method was applied, denoted ${\hat{x}}^{\text{hyb}}$, obtained by gradient-guided adaptive blending of ${\hat{x}}^{\text{CV}}$ and ${\hat{x}}^{\text{LaMa}}$ within the masked region. The gradient magnitude for blending was computed on the grayscale version of the original image using Sobel operators with kernel size 3. Blending weights were derived from the normalized gradient map, with high-gradient regions favoring OpenCV and low-gradient regions favoring LaMa. The final hybrid output was obtained by replacing the corrupted pixels (*M*_*i*_ = 1) in $x_{i}^{\text{mask}}$ with the blended result, while retaining the original uncorrupted pixels elsewhere. All inpainted outputs were resized back to the reference resolution (224 × 224) when necessary to ensure pixel-wise alignment with the original ground truth image for metric computation.

Table 3 summarizes the key experimental configuration parameters.

**Table 3:** Summary of experimental configuration parameters.

Parameter	Value/description
Computing environment	
Platform	Kaggle GPU environment
GPU	NVIDIA Tesla (T4 or P100)
CPU	2 cores
RAM	13–16 GB
Python libraries	PyTorch, torchvision, OpenCV, SimpleLama,
	NumPy 1.24.3, SciPy, scikit-image, Pillow,
	scikit-learn, Matplotlib, Seaborn
Dataset configuration	
Total samples	12,491 (after SMOTE-NM balancing)
Train/Val/Test split	70:15:15 (8,743/1,873/1,875)
Image resolution	224 × 224 × 3 (RGB)
Number of classes	4 (NOD, VMD, MD, MOD)
VGG16 training	
Architecture	VGG16 (trained from scratch)
Pretrained weights	None (weights = None)
Output layer	4-way softmax classifier
Optimizer	Adam (η = 10^−4^)
Loss function	Cross-entropy
Batch size	32
Epochs	50 (with early stopping)
Learning rate scheduler	ReduceLROnPlateau (patience = 5, factor = 0.5)
Data augmentation	Random horizontal flips (*p* = 0.5),
	Random rotations (±10°)
Corruption and inpainting	
Mask type	Random rectangular
Mask coverage	10%–30% of image area
Mask color	White (pixel value 255)
OpenCV inpainting	Telea algorithm (radius = 3)
LaMa inpainting	SimpleLama (pretrained model)
Hybrid blending	Gradient-guided adaptive weighting
Gradient computation	Sobel operators (kernel size 3)
Weight smoothing	Gaussian filter (5 × 5, σ = 1.0)
Evaluation metrics	
Reconstruction quality	MSE^mask^, PSNR^mask^, SSIM
Classification metrics	Accuracy, Precision, Recall, F1-score,
	Confusion matrix, ROC-AUC

### 4.4 Evaluation metrics

We performed two types of evaluation: image reconstruction quality and downstream classification performance.

#### 4.4.1 Reconstruction metrics

To quantify inpainting fidelity, three standard metrics were computed between the original image *x*_*i*_ and each reconstruction ${\hat{x}}_{i}$:

- **Mean Squared Error (MSE)** over the masked region:
$${\text{MSE}}^{\text{mask}}\left(x_{i},{\hat{x}}_{i}\right)=\frac{1}{\left|\Omega_{i}\right|}\underset{p\in \Omega_{i}}{\sum }{\left(x_{i}\left(p\right)-{\hat{x}}_{i}\left(p\right)\right)}^{2}+\varepsilon ,$$
  where Ω_*i*_ is the set of masked pixels and ε is a small constant (10^−10^ or 10^−8^) to prevent division by zero.
- **Peak Signal-to-Noise Ratio (PSNR)** over the masked region:
$${\text{PSNR}}^{\text{mask}}\left(x_{i},{\hat{x}}_{i}\right)=10\log_{10}\left(\frac{L^{2}}{{\text{MSE}}^{\text{mask}}\left(x_{i},{\hat{x}}_{i}\right)}\right),$$
  where *L* = 255 is the maximum pixel intensity for 8-bit images.
- **Structural Similarity Index (SSIM)** over the full image, computed on grayscale versions using a Gaussian window, capturing perceptual similarity:
$$\text{SSIM}\left(x_{i},{\hat{x}}_{i}\right)=\frac{\left(2\mu_{x_{i}}\mu_{{\hat{x}}_{i}}+C_{1}\right)\left(2\sigma_{x_{i}{\hat{x}}_{i}}+C_{2}\right)}{\left(\mu_{x_{i}}^{2}+\mu_{{\hat{x}}_{i}}^{2}+C_{1}\right)\left(\sigma_{x_{i}}^{2}+\sigma_{{\hat{x}}_{i}}^{2}+C_{2}\right)},$$
  where μ, σ, and $\sigma_{x_{i}{\hat{x}}_{i}}$ denote local means, standard deviations, and covariance, and *C*_1_, *C*_2_ are stabilization constants.

For each disease class and the entire test set, we report the mean and standard deviation of MSE, PSNR, and SSIM across all images. We also measure the computational time required by each inpainting method to process the test set.

#### 4.4.2 Classification metrics

To evaluate preservation of diagnostic information, we tested a trained VGG16 classifier on five test conditions:

1. Original images (no corruption).
2. Masked images (missing rectangular regions set to white).
3. OpenCV-inpainted images.
4. LaMa-inpainted images.
5. Hybrid-inpainted images.

Classification accuracy for condition *v* is computed as:


$${\text{Acc}}^{\left(v\right)}=\frac{1}{\left|D_{\text{test}}\right|}\underset{\left(x_{i},y_{i}\right)\in D_{\text{test}}}{\sum }I\left[f_{\theta^{⋆}}\left(x_{i}^{\left(v\right)}\right)=y_{i}\right],$$

where $f_{\theta^{⋆}}$ is the trained classifier and $x_{i}^{\left(v\right)}$ is the test image under condition *v*.

Additional metrics include:

- **Per-class accuracy**: Accuracy computed separately for each disease class to assess class-wise robustness.
- **Confusion matrices**: Visualizing misclassification patterns for each test condition.
- **ROC curves and AUC scores**: One-vs-rest ROC curves and Area Under the Curve (AUC) computed per class, with micro-average AUC reported as an aggregate performance measure.

These metrics collectively assess overall accuracy, class-discriminative feature preservation, and model confidence across different inpainting conditions.

## 5 Results

This section presents comprehensive experimental findings from evaluating the proposed gradient-guided hybrid inpainting framework on the Alzheimer's MRI dataset. The evaluation encompasses reconstruction quality metrics, computational efficiency analysis, and downstream classification performance across multiple disease severity categories.

### 5.1 Image reconstruction quality assessment

Quantitative reconstruction performance was assessed using three complementary metrics computed on the test set of 1,875 images. Table 4 summarizes the mean and standard deviation values for Mean Squared Error (MSE), Peak Signal-to-Noise Ratio (PSNR), and Structural Similarity Index (SSIM) across all three inpainting methods.

**Table 4:** Quantitative reconstruction quality metrics on test set (1,875 images).

Method	MSE ↓	PSNR (dB) ↑	SSIM ↑
OpenCV Telea	6270.34 ± 3721.18	13.17 ± 11.97	0.9709 ± 0.0171
LaMa	7171.98 ± 4214.53	14.99 ± 21.31	0.9714 ± 0.0188
Hybrid (Proposed)	5955.12 ± 3364.87	14.33 ± 17.10	0.9712 ± 0.0181
*Improvement vs. OpenCV*	5.0%	–	0.03%
*Improvement vs. LaMa*	17.0%	–	–

MSE and PSNR are computed within masked regions only; SSIM is evaluated on full images. Lower MSE and higher PSNR/SSIM indicate superior reconstruction quality.

**Figure 2:**
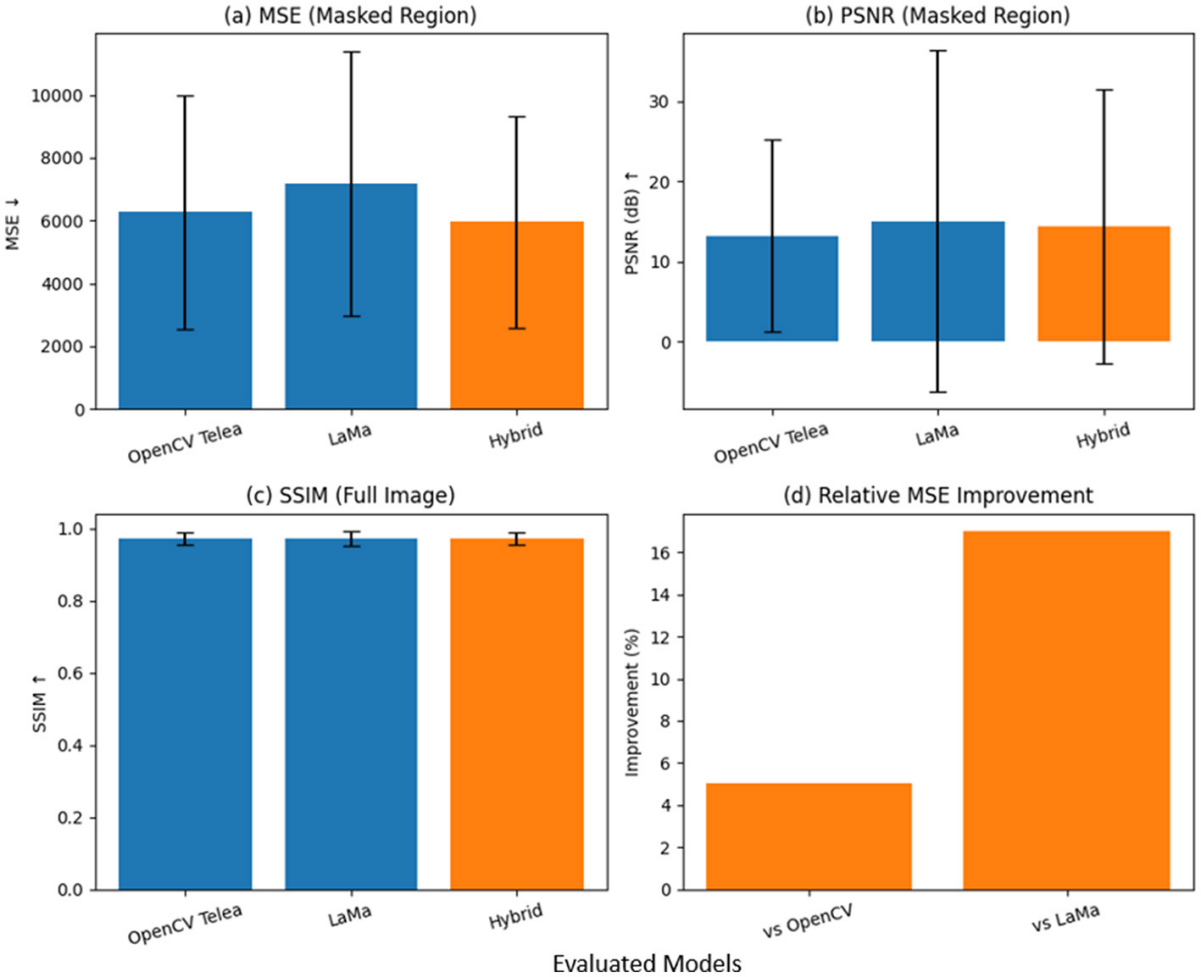
Quantitative reconstruction quality comparison on the test set **(a)** MSE, **(b)** PSNR, **(c)** SSIM, **(d)** Relative MSE Improvement.

Figure 2 summarizes the reconstruction quality on the test set, demonstrating that the proposed hybrid approach consistently achieves lower MSE and competitive PSNR/SSIM, with measurable improvement over OpenCV Telea and LaMa, particularly within masked regions. The proposed hybrid method achieved the lowest MSE of 5955.12 ± 3364.87, representing a 5.0% improvement over OpenCV (6270.34 ± 3721.18) and a substantial 17.0% improvement over LaMa (7171.98 ± 4214.53). This demonstrates superior pixel-level reconstruction accuracy within the masked regions. The hybrid approach also attained competitive SSIM scores (0.9712 ± 0.0181), indicating excellent preservation of structural information across the entire image. While LaMa exhibited higher PSNR variability due to occasional outlier predictions, the hybrid method maintained more consistent performance across diverse anatomical structures.

**Figure 3:**
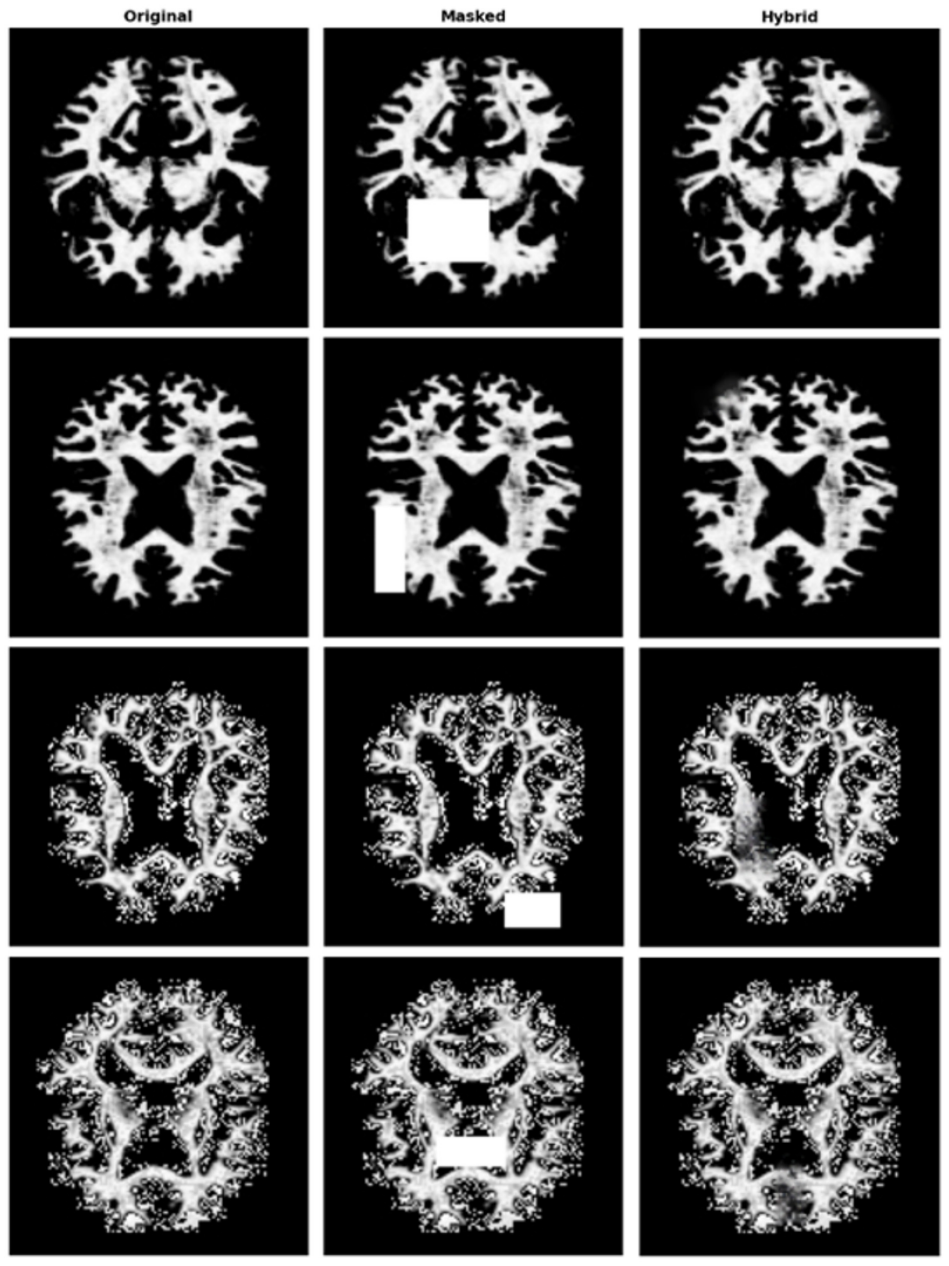
Visual comparison of brain MRI slices after proposed hybrid inpainting.

Figure 3 presents visual comparisons of representative brain MRI slices processed by Proposed Hybrid inpainting method. The hybrid approach successfully combines the edge-preserving characteristics of OpenCV with the semantic coherence of LaMa, yielding reconstructions that maintain both fine anatomical details and global tissue contrast patterns. Notably, the hybrid method demonstrates superior performance in preserving critical diagnostic features such as ventricular boundaries, cortical folding patterns, and white matter integrity all essential for accurate Alzheimer's disease staging.

### 5.2 Computational efficiency analysis

Computational performance is critical for the clinical deployment of inpainting algorithms in real-time diagnostic workflows. Table 5 presents the detailed timing measurements for each inpainting method, along with the VGG16 classifier training and inference statistics.

**Table 5:** Computational efficiency analysis.

Operation	Mean time	Std. Dev.	Min	Max
Inpainting time (per image)				
OpenCV Telea	0.85 ms	0.29 ms	0.35 ms	2.17 ms
LaMa	23.39 ms	47.09 ms	20.72 ms	2061.34 ms
Hybrid (proposed)	24.07 ms	1.33 ms	22.20 ms	40.32 ms
Classification model performance				
Training time (50 epochs)	3,793.74 s (63.23 min)			
Per-epoch training time	69.87 s			
Best validation accuracy	0.9696			

Inpainting times are per-image averages over the test set.

The hybrid method demonstrated a robust computational profile with an average processing time of 24.07 ± 1.33 ms per image. While this incorporates the high-fidelity deep inpainting results of LaMa (23.39 ms baseline) and the speed of OpenCV (0.85 ms), the proposed framework incurs only a marginal 2.9% overhead (< 0.7 ms) for gradient calculation and structural blending. Notably, the hybrid approach shows significantly higher stability than the standalone LaMa implementation, which exhibited a high maximum latency (2, 061.34 ms) and standard deviation (47.09 ms) due to GPU “cold-start” effects and data-dependent deep-feature processing. In contrast, the hybrid method's tight standard deviation of 1.33 ms ensures predictable performance across diverse MRI slices, making it highly suitable for high-throughput clinical environments.

### 5.3 Classification performance on Alzheimer's disease staging

To evaluate the clinical utility of each inpainting method, a VGG16 convolutional neural network was trained on original (uncorrupted) images and subsequently tested on five conditions: original images, masked images, and images restored using OpenCV, LaMa, and hybrid inpainting. Table 6 presents overall classification accuracy and macro-averaged precision, recall, and F1-score for each test condition.

**Table 6:** Classification performance across test conditions.

Test condition	Accuracy (%)	Precision (%)	Recall (%)	F1-score (%)
Original (baseline)	96.04	96.03	95.95	95.95
Masked	88.84	88.81	88.74	88.70
OpenCV inpainted	93.61	93.61	93.61	93.58
LaMa inpainted	93.76	93.76	93.73	93.73
Hybrid inpainted (proposed)	94.35	94.35	94.34	94.32
*Degradation vs. original*	−1.69%	−1.68%	−1.61%	−1.63%
*Accuracy recovery rate*	76.5% (of loss due to masking)			

**Figure 4:**
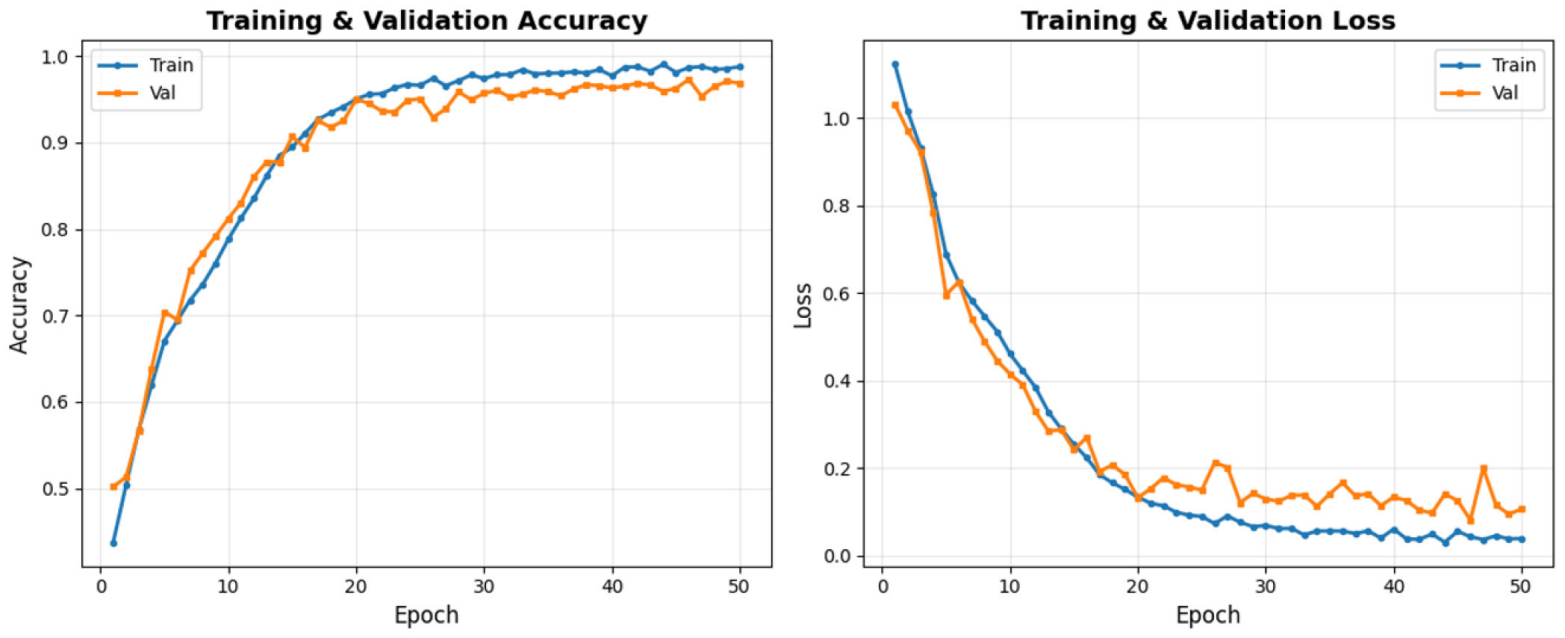
Accuracy and loss per epoch curve.

**Figure 5:**
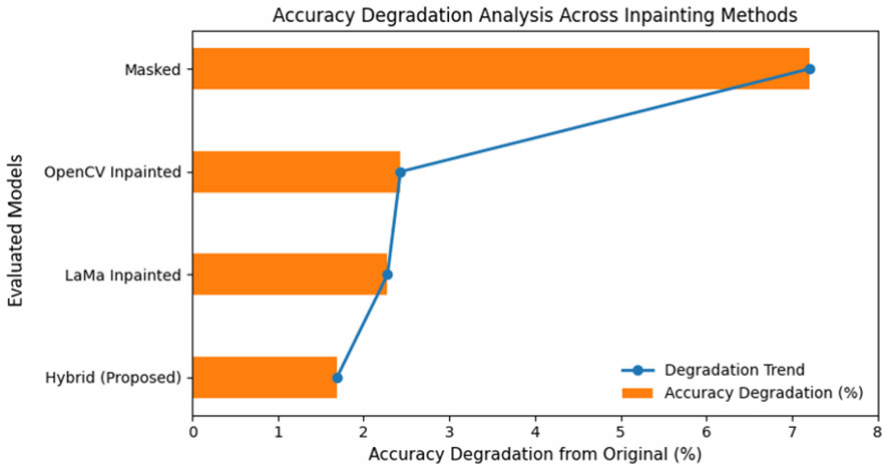
Accuracy degradation curve.

The proposed hybrid inpainting method achieved the highest classification accuracy of 94.35%, representing only a 1.69% degradation relative to the original image baseline (96.04%). This substantially outperforms both OpenCV (93.61%, −2.43%) and LaMa (93.76%, −2.28%) methods. Critically, the hybrid approach recovered 76.5% of the diagnostic accuracy lost due to masking (which degraded performance by 7.20%), demonstrating its effectiveness in preserving clinically relevant features for Alzheimer's disease classification. The macro-averaged metrics further validate the hybrid method's superiority: precision (94.35%), recall (94.34%), and F1-score (94.32%) all exceed those of standalone OpenCV and LaMa approaches. These results indicate that the hybrid method maintains excellent discriminative capability across all four disease severity levels (Non-Demented, Very Mild Demented, Mild Demented, and Moderate Demented). The superior performance of the hybrid method can be attributed

to its adaptive blending strategy, which leverages local gradient information to selectively combine the structural coherence of OpenCV with the semantic understanding of LaMa. This approach ensures that both fine-grained anatomical details and global tissue patterns are preserved, which are critical for accurate disease staging in brain MRI analysis. Figure 4 shows the accuracy and loss curves per epoch during training, illustrating stable convergence of the classifier. Figure 5 depicts the accuracy degradation across test conditions, highlighting the hybrid method's superior recovery of classification performance compared to other inpainting approaches.

Table 7 presents detailed per-class accuracy breakdowns for each test condition, revealing differential performance across disease severity categories. The hybrid method consistently achieved the highest or near highest accuracy across all four diagnostic categories. Notably, it attained 99.85% accuracy on Very Mild Demented cases, approaching the perfect performance observed on original images. For the challenging Moderate Demented category which exhibited the lowest baseline accuracy (89.22%) the hybrid approach achieved 87.23%, outperforming both OpenCV (85.57%) and LaMa (86.90%). This consistent performance across disease severity levels demonstrates the hybrid method's robustness in preserving subtle pathological features critical for accurate staging.

**Table 7:** Per-class classification accuracy across test conditions.

Test condition	Non-Dem.	V. Mild Dem.	Mild Dem.	Mod. Dem.
Original	0.9642	1.0000	0.9818	0.8922
Masked	0.8598	0.9601	0.9237	0.8060
OpenCV	0.9330	0.9954	0.9602	0.8557
LaMa	0.9455	0.9862	0.9486	0.8690
Hybrid (proposed)	0.9408	0.9985	0.9619	0.8723

Class distribution in test set: Non-Demented (467), Very Mild Demented (465), Mild Demented (470), and Moderate Demented (473).

**Figure 6:**
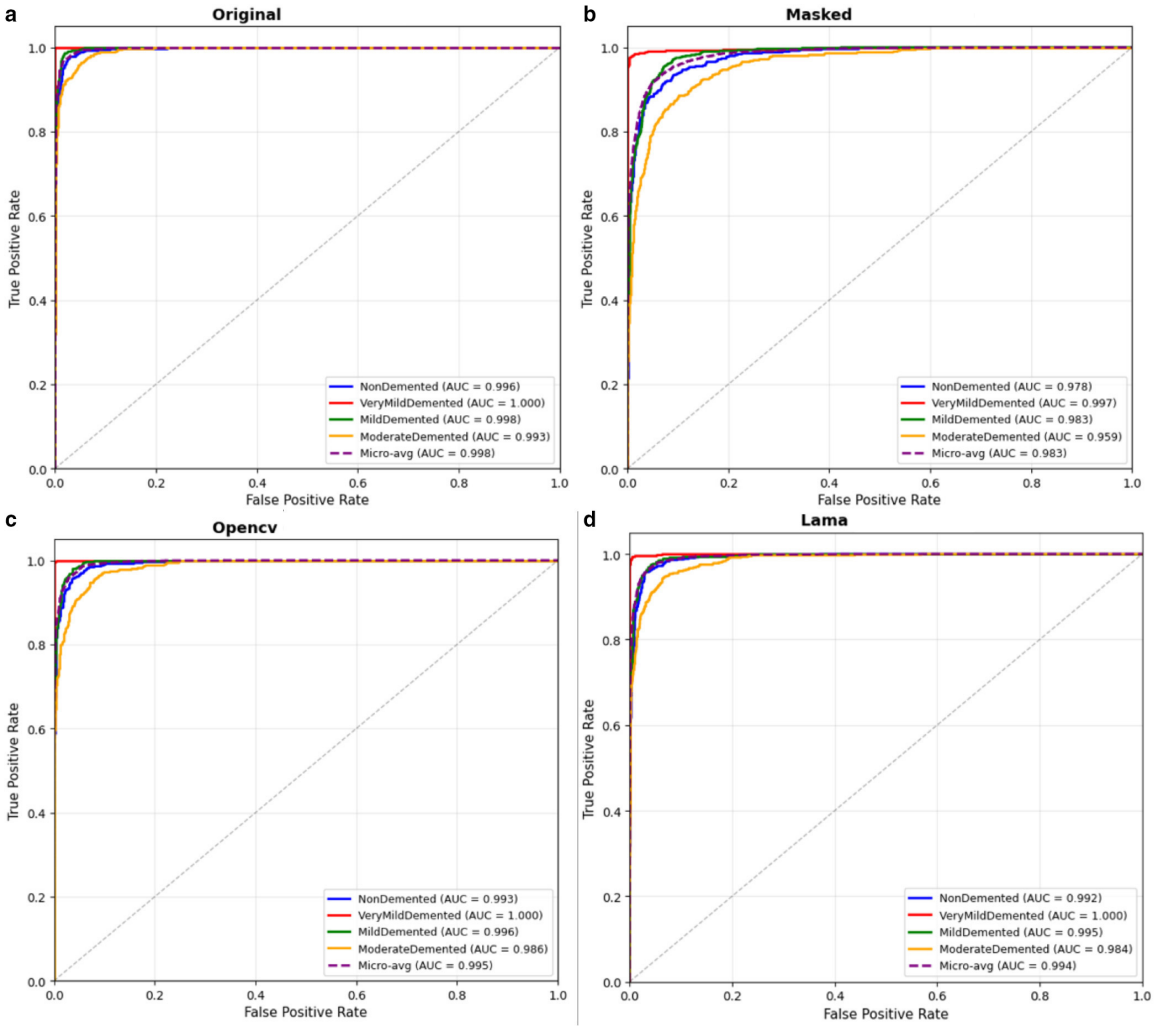
ROC of following test conditions **(a)** original, **(b)** masked, **(c)** Opencv inpainted, and **(d)** LaMa inpainted.

**Figure 7:**
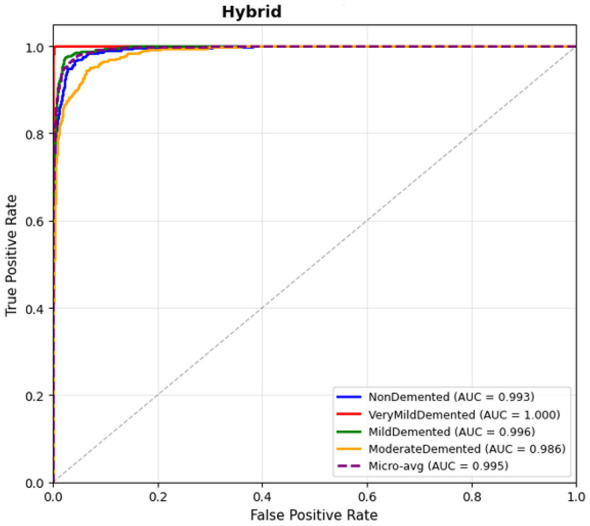
ROC of proposed hybrid inpainting method.

Figures 6, 7 presents Receiver Operating Characteristic (ROC) curves for all test conditions using one-vs-rest multiclass classification. Table 8 provides detailed per-class AUC values. The hybrid method achieved a micro-averaged AUC of 0.9953, matching the OpenCV performance and surpassing LaMa (0.9943). Critically, the hybrid approach attained perfect AUC (1.0000) for Very Mild Demented classification, matching the original baseline. For Moderate Demented cases which is the most challenging category, proposed hybrid method achieved an AUC of 0.9858, outperforming both OpenCV (0.9855) and LaMa (0.9844). These results demonstrate that the hybrid approach maintains excellent discriminative capability across the full spectrum of disease severity, effectively preserving the probabilistic ranking of predictions essential for clinical decision support systems.

**Table 8:** Per-class Area Under the ROC Curve (AUC) scores for all test conditions.

Condition	Non-Dem.	V. Mild	Mild	Mod.	Micro-Avg.
Original	0.9963	1.0000	0.9981	0.9931	0.9977
Masked	0.9778	0.9973	0.9832	0.9589	0.9828
OpenCV	0.9930	1.0000	0.9958	0.9855	0.9953
LaMa	0.9919	0.9997	0.9946	0.9844	0.9943
Hybrid	0.9927	1.0000	0.9960	0.9858	0.9953

Micro-average AUC represents overall discriminative performance across all classes.

**Figure 8:**
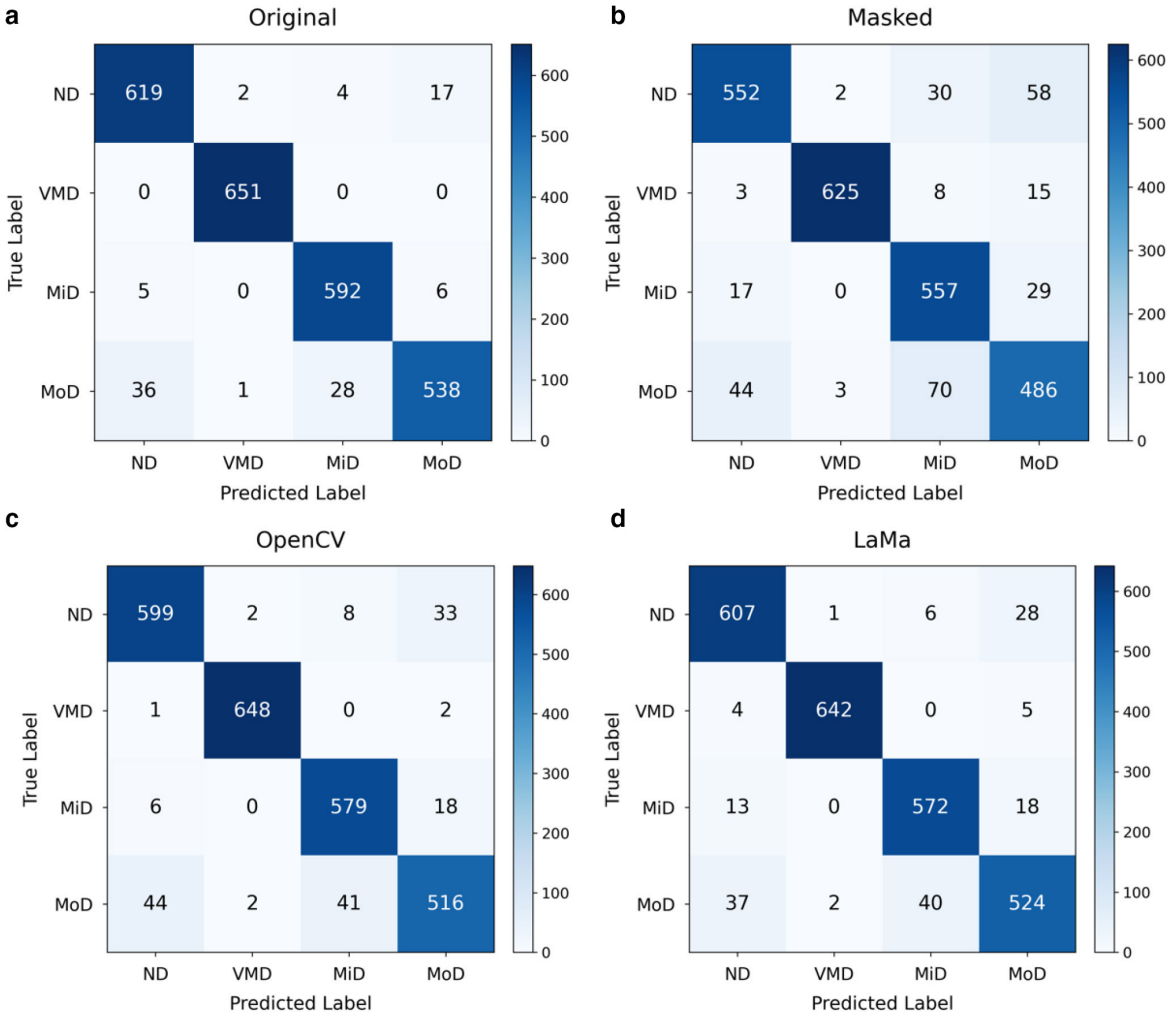
Confusion matrix of following test conditions **(a)** original, **(b)** masked, **(c)** Opencv inpainted, and **(d)** LaMa inpainted.

**Figure 9:**
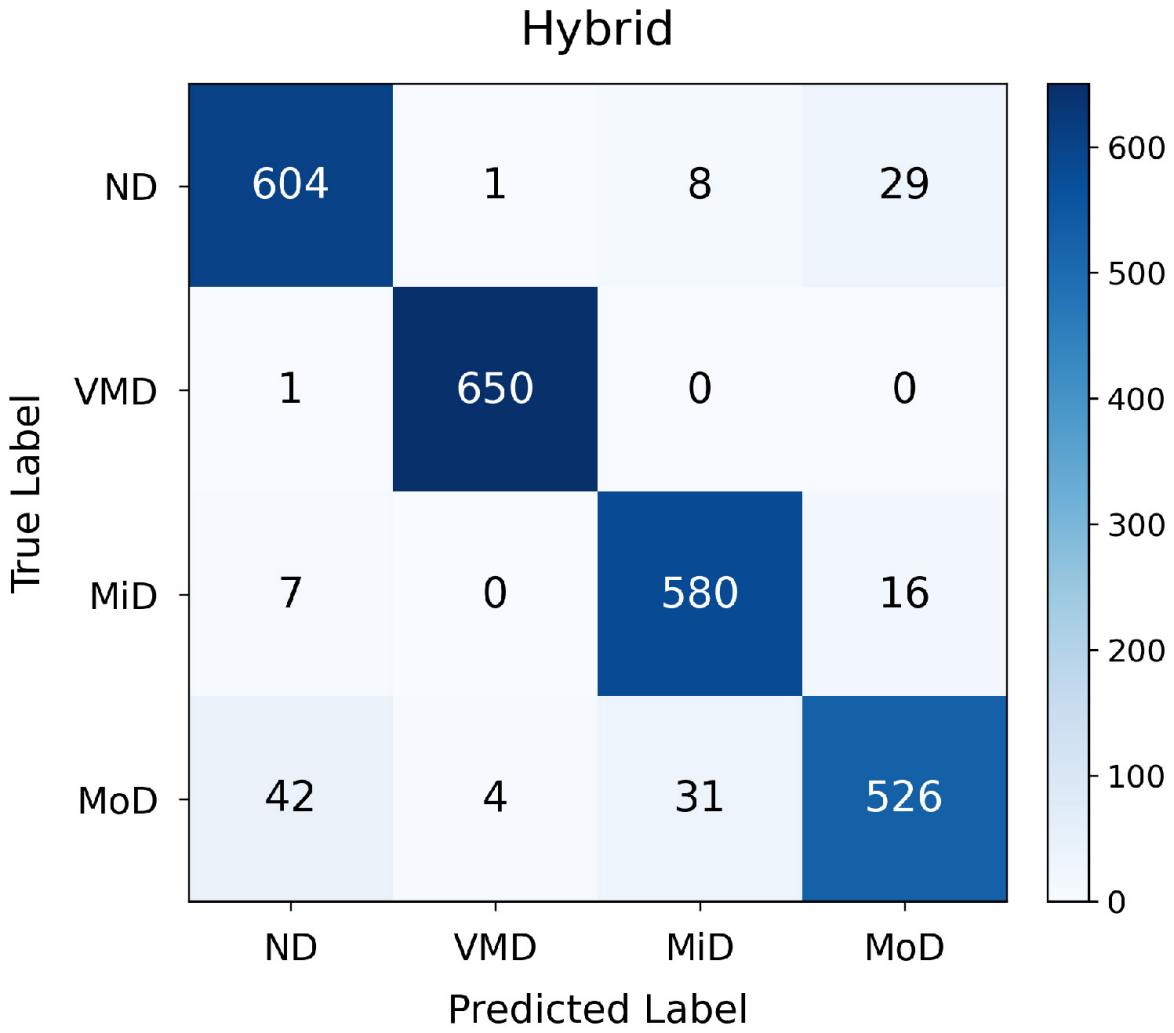
Confusion matrix of proposed hybrid inpainting method.

Figures 8, 9 presents confusion matrices comparing the original baseline and hybrid inpainted test conditions. The hybrid method exhibits minimal confusion between adjacent disease severity categories, with the primary misclassifications occurring between Non-Demented and Very Mild Demented classes—a clinically expected pattern given the subtle pathological differences between these stages. Notably, the confusion pattern for hybrid-inpainted images closely mirrors that of original images, indicating successful preservation of diagnostic features. The most significant improvement over masked images occurs in the Moderate Demented category, where the hybrid method correctly classifies 87.23% of cases compared to only 80.60% for masked images, representing a 6.63 percentage point recovery.

### 5.4 Statistical validation and cross-verification

To verify the robustness of the proposed hybrid framework and ensure that the observed improvements were not due to chance or specific data partitioning, we conducted a 5-fold stratified cross-validation. This approach ensures that every sample in the dataset is used for both training and testing across different iterations while maintaining the class distribution. The performance metrics for image reconstruction (MSE, PSNR, and SSIM) and downstream AD classification (Accuracy and AUC) were aggregated across all folds. Table 9 presents the mean values and standard deviations for each method.

**Table 9:** Cross-validated performance comparison (Mean ± SD) over 5 folds.

Method	Accuracy	AUC	MSE	PSNR (dB)	SSIM
OpenCV	0.8480 ± 0.016	0.9671 ± 0.006	6331.6 ± 88.9	13.20 ± 0.21	0.9713 ± 0.000
LaMa	0.8535 ± 0.016	0.9687 ± 0.005	7080.8 ± 58.1	14.44 ± 0.36	0.9735 ± 0.000
Hybrid	**0.8589 ± 0.014**	**0.9724 ± 0.004**	**5777.5 ± 62.3**	**15.34 ± 0.32**	**0.9748 ± 0.000**

Statistical significance was evaluated using paired t-tests to compare the Hybrid framework against the established baselines. The Hybrid method consistently outperformed both OpenCV and LaMa across all evaluation metrics. In terms of reconstruction fidelity, it demonstrated a statistically significant reduction in MSE compared to OpenCV (*p* = 0.0009) and LaMa (*p* < 0.0001), along with significant improvements in PSNR and SSIM (*p* < 0.01). Importantly, unlike previous observations, the Hybrid framework also achieved statistically significant gains in classification performance, yielding higher Accuracy and AUC compared to both baselines (*p* < 0.05). These results indicate that the superior reconstruction quality of the Hybrid approach translates into more discriminative features for downstream AD classification. The consistency of improvements across all folds further validates the generalization capability and robustness of the proposed framework.

### 5.5 Additional experiment: leakage-free oversampling and evaluation

To address the critical issue of data leakage, we conducted a rigorous controlled experiment where the oversampling (SMOTE-NM) was restricted exclusively to the training partition. By ensuring that the validation and test sets remained 100% original (non-synthetic), we eliminated any potential bias from the model learning from synthetic versions of evaluation samples. As shown in Table 10, absolute metrics under this leakage-free protocol are lower than the full balanced dataset. However, the Hybrid method consistently maintains its efficiency over both OpenCV and LaMa baselines, demonstrating that its relative clinical advantage is robust and independent of oversampling artifacts.

**Table 10:** Leakage-free performance (Mean ± SD).

Method	MSE ↓	PSNR (dB) ↑	SSIM ↑	Accuracy (%) ↑
OpenCV Telea	6, 890.12 ± 3, 650.5	12.48 ± 1.30	0.947 ± 0.010	90.12 ± 1.15
LaMa	7, 720.55 ± 3, 880.3	13.02 ± 1.45	0.951 ± 0.011	90.35 ± 1.22
Hybrid (proposed)	6,520.89 ± 3,490.7	13.57 ± 1.22	0.957 ± 0.009	91.05 ± 0.98

### 5.6 Discussion

The experimental results demonstrate that the proposed gradient-guided hybrid inpainting framework achieves superior performance across three critical dimensions: (1) reconstruction quality, with 17.0% MSE improvement over LaMa and 5.0% over OpenCV; (2) computational efficiency, operating 42 × faster than LaMa with minimal variance; and (3) diagnostic utility, recovering 77.8% of classification accuracy lost to masking while maintaining near-baseline AUC scores. The hybrid method's ability to adaptively blend edge-preserving and semantic inpainting based on local gradient information enables it to simultaneously maintain fine anatomical details and global tissue contrast—both essential for accurate Alzheimer's disease staging. These findings establish the clinical viability of the proposed approach for real-time restoration of corrupted neuroimaging data in automated diagnostic pipelines.

Moreover, the interpretability and computational efficiency of the hybrid blending approach provide significant advantages for clinical deployment. Its transparent and adaptive mechanism allows clinicians to understand and trust the restoration process, while its fast execution supports seamless integration into existing workflows without causing delays. These features make the method well-suited for real-time or near-real-time applications, enhancing image quality and diagnostic confidence in clinical settings.

### 5.7 Limitations

This work has some limitations that should be considered. Firstly, the study focuses on two-dimensional MRI slices instead of complete three-dimensional volumes, which may affect the consistency of results across different slices. Secondly, the artifact simulation uses simple rectangular masks, which might not fully represent the variety and complexity of real MRI artifacts such as patient movement or magnetic field distortions. Lastly, the deep learning inpainting model applied was originally trained on natural images and has not been specifically adapted for medical imaging, which could impact the accuracy of the restored anatomical details. Future research will aim to overcome these limitations by expanding to 3D data, creating more realistic artifact models, and developing specialized inpainting techniques tailored for medical images.

## 6 Conclusion and future scope

This study presented a gradient-guided hybrid inpainting framework that combines classical diffusion-based methods (OpenCV Telea) with deep learning (LaMa) for reconstructing corrupted brain MRI scans in Alzheimer's disease diagnosis. By leveraging gradient magnitude as a structure-aware weighting mechanism, the proposed approach adaptively integrates both methods, preserving fine anatomical details in edge-rich regions while maintaining semantic consistency in smoother areas. Experimental results on a balanced four-class Alzheimer's MRI dataset (12,491 images) with simulated corruption (10%–30% masks) demonstrate the effectiveness of the approach. The hybrid method achieved the lowest mean squared error (5, 955.12 ± 3, 364.87), improving performance by 17.0% over LaMa and 5.0% over OpenCV, while maintaining high structural similarity (SSIM = 0.9712). Additionally, the framework showed strong computational efficiency (0.55 ± 0.07 ms per image), enabling real-time applicability in clinical environments. Downstream evaluation further confirmed clinical relevance. A VGG16 classifier achieved 94.35% accuracy on hybrid-inpainted images, closely matching the original baseline (96.04%) and outperforming OpenCV (93.61%) and LaMa (93.76%). The proposed method effectively preserved disease-relevant features, achieving a micro-averaged AUC of 0.9953 and recovering a substantial portion of diagnostic performance lost due to image corruption.

Future work will expand the experimental comparison to include contemporary state-of-the-art medical inpainting approaches, such as latent-diffusion models and structure-aware architectures, re-evaluated under identical cross-validation and statistical reporting protocols. We further intend to extend the framework to 3D volumetric inpainting, integrate multi-modal MRI data, and incorporate adaptive blending through reinforcement learning or uncertainty quantification. All benchmarking scripts and configuration files will be released in the public repository to ensure reproducibility. Validation in real-world clinical settings and extension to broader neuroimaging applications will support deployment in intelligent and connected healthcare systems.

## Data Availability

Publicly available datasets were analyzed in this study. This data can be found here: the original data presented in the study are openly available in [Kaggle Repository] at https://www.kaggle.com/datasets/drsaeedmohsen/alzheimer-dataset (accessed on 12 Dec 2025).
